# Serum Angiopoietin-2 and β-hCG as Predictors of Prolonged Uterine Bleeding after Medical Abortion in the First Trimester

**DOI:** 10.1371/journal.pone.0063755

**Published:** 2013-05-16

**Authors:** Maofeng Wang, Junqing Chen, Jun Ying, Jiong Yu, Bifei Huang, Zhaoxiang Ren, Xianyu Wang, Qiaoqiao Guo, Yunlai Wang, Liuyi Qiu, Hongsheng Yu, Rugen Wan

**Affiliations:** 1 Department of Clinical Laboratory Medicine, Affiliated Dongyang Hospital of Wenzhou Medical College, Dongyang, Zhejiang, People’s Republic of China; 2 Department of Gynecology, Affiliated Dongyang Hospital of Wenzhou Medical College, Dongyang, Zhejiang, People’s Republic of China; 3 School of Laboratory Medicine, Wenzhou Medical College, Wenzhou, Zhejiang, People’s Republic of China; 4 State Key Laboratory for Diagnosis and Treatment of Infectious Diseases, The First Affiliated Hospital, School of Medicine, Zhejiang University, Hangzhou, Zhejiang, People’s Republic of China; 5 Pathology Center, Affiliated Dongyang Hospital of Wenzhou Medical College, Dongyang, Zhejiang, People’s Republic of China; 6 Department of Ultrasonography, Affiliated Dongyang Hospital of Wenzhou Medical College, Dongyang, Zhejiang, People’s Republic of China; Ottawa Hospital Research Institute, Canada

## Abstract

**Objective:**

The combination of mifepristone and misoprostol is an established method for induction of early first trimester abortion, but there is no consensus about the best evaluation of treatment outcome. We evaluate serum Angiopoietin-2(Ang-2) and β human chorionic gonadotropin (β-hCG) in women who had undergone a medical abortion as markers of prolonged uterine bleeding (PUB).

**Methods:**

Prospective trial involving 2843 women attending an gynecology outpatient clinic who following a medical abortion with mifepristone and misoprostol, the study cohort was divided into women with duration of uterine bleeding >14 days (PUB) and women with duration of uterine bleeding ≤14 days (normal uterine bleeding, NUB). Serum determinations of Ang-2 levels by ELISA and β-hCG levels by electrochemiluminiscence immunoassay. Receiver Operating Characteristics (ROC) analyses were calculated and plotted for the diagnostic accuracy of serum β-hCG and Ang-2 concentration to discriminate PUB and NUB.

**Results:**

Baseline characteristics for both groups were similar, Only duration of bleeding showed a significant difference between the PUB group and NUB group. Ang-2 serum levels moderately correlated with serum β-hCG levels with statistically significant correlation coefficients of 0.536. Serum β-hCG and Ang-2 levels on day 7 and on day 14 after medical abortion were signifcantly higher in PUB group than in NUB group. Plotted as ROC curves, β-hCG area under curve (AUC) was 0.65 (95% CI, 0.53–0.76) on day 7, rising to AUC = 0.83 (95% CI, 0.75–0.92) on day 14. Using Ang-2 on day 7 and day 14 as predictive parameter resulted in an analogous AUC (AUC = 0.61 on day 7, AUC = 0.78 on day 14).

**Conclusions:**

Both parameters are clinically useful as a diagnostic test in predicting PUB after medical abortion, and can be helpful in uncertain clinical situations, but should be considered as supplementary to a general clinical evaluation.

## Introduction

The World Health Organization (WHO) reported that there were 43.8 million induced abortions in 2008 globally [1],there are about 9 million induced abortions in China every year and the figure is increasing annually [2]. First trimester abortion with medications rather than surgery is widely used throughout the world, Medical abortion has the potential to expand abortion services, where surgical services are limited, and to expand women’s choice of abortion method and experience [3]. Drugs such as mifepristone and misoprostol for medical abortion are now widespread throughout the world [4]. Many studies have shown that these drugs are highly efficacious in terminating unwanted pregnancies in the first or second trimesters and even in managing complications of abortion [4], [5], [6], [7]. Furthermore, these drugs have been included in the WHO’s Essential Drugs List and recommended as a key for managing unwanted pregnancy and improving abortion safety [5]. Most hospitals in China currently use repeated small doses of 25 mg (total: 150 mg) of mifepristone in combination with misoprostol as standard for early medical abortion in women ≤49 days pregnant [8]. The most common significant adverse event or outcome reported was ongoing intrauterine pregnancy, occurring in 0.5% of all medical abortion procedures. However, this regimen usually has a significantly longer bleeding time and a greater amount of hemorrhage than surgical abortion [4]. Generally, vaginal bleeding after a complete medical abortion lasts for about 14–15 days, on average, as compared to <1 week after vacuum aspiration. However, a small section of women experience persistent bleeding for three weeks or even longer [8]. Although the quantity of bleeding is usually lighter than menses or just light spotting, PUB poses not only health problems, but also acceptability problems. Expected duration of bleeding and factors that predict prolonged bleeding associated with a medical abortion are important for patient counseling. It is also a concern for the attending doctor, as prolonged bleeding can be a sign of an incomplete abortion.

Incomplete medical abortion may increase the risk of infection and is furthermore associated with discomfort as persistent or recurrent bleeding and pain. Early identification of women with Incomplete medical abortion would allow early intervention. Attempts to discover diagnostically relevant serum markers that prediction of failure after medical abortion, has led to the investigation of various factors [9], [10], [11], [12]. However, none of these factors appears to be capable of identifying cases with a definite unwanted outcome. The absolute and/or relative β-hCG be higher in failures than successes, and could perhaps be an indicator of failure after medical abortion [9], [11]. An important, yet underappreciated family of Angiogenic proteins is that of the Angiopoietin (Ang) family, which has been shown to be critically involved in vasculature development and Angiogenesis, especially in the female reproductive tract [13], [14], [15]. Angs are intimately involved in the process of placental maturation and growth from early pregnancy [16], [17], [18], [19]. Angiopoietin-1 (Ang-1) and Ang-2 are critical regulators with different functions of vascular development and Angiogenesis [20]. Ang-2 are expressed in the early placenta in normal and pathological pregnancy, which has been explored in numerous studies [18], [21], [22]. Ang-2 synthesis and actions in the fetal–placental unit could be reflected in the maternal serum concentrations as has been reported for other Angiogenic factors, such as placental growth factor and soluble vascular growth factor receptor-1 [22]. The mRNA of Ang-2 and the expressions of Ang-2 proteins were increased in late first-trimester decidua basalis. These increased levels of angiogenic factors suggest increased angiogenic activity at the implantation site as gestation progresses [23]. Collectively, these data support the notion that maternal serum Ang-2 levels of healthy pregnancies could be altered after medical abortion. Thus, Ang-2 appear to be appealing Angiogenic candidate marker for prediction of PUB after medical abortion. However, the prognostic significance of simultaneous serological measurements of β-hCG and Ang-2 in First trimester medical abortion has not been studied so far.

With this background, we wanted to compare the β-hCG and Ang-2 after medical abortion 2 weeks in PUB and NUB groups, and to analyse the prognostic value of the two variables.

## Materials and Methods

### Ethics Statement

Ethical approval for this study was granted from the Affiliated Dongyang Hospital of Wenzhou Medical College Human Investigation Ethics Committee and all women gave written informed consent for enrolment into the study.

### Subjects and Sample Collection

Women with non-complicated singleton pregnancies following a medical abortion were recruited. Entry criteria included: (1) age 20–40 years; (2) normal menstrual cycles; (3) a singleton intrauterine pregnancy not exceeding 49 days’ gestation (based on the onset of the last menstrual period, bimanual examination and ultrasound); (4) the women had neither genital inflammation nor neoplasms, and had not received exogenous hormones within the previous six months. In accordance with working routines, The first visit was for clinical and ultrasonographic assessment to confirm pregnancy and determine gestational age by measurement of crown–rump length. Medical abortion clients received mifepristone (50 mg) orally on their first visit, then 25 mg every 12 hours at home (total:150 mg). At the second visit, 48 hours later, women received oral misoprostol (600 mg) and remained in hospital under observation for four hours. On 14 day, the women returned for the final follow-up visit. If they had aborted, they followed the scheduled treatment protocol; if not, they were examined again at the clinic the same day. According to the results of the examination, an individual follow-up plan was formed. 44 women with duration of bleeding>14 days after a medical abortion were enrolled in the PUB group. 1∶1 sample size will achieve the highest statistical power, so a cohort of 44 women with total duration of bleeding ≤14 days after a medical abortion would be adequate for statistically significant comparisons with PUB group. All subjects were recruited at Affiliated Dongyang Hospital of Wenzhou Medical College between 1 January and 20 December, 2012. Blood samples were drawn prior to mifepristone received for baseline data, on day 7 and day 14, blood samples were drawn again for β-hCG and Ang-2 measurement. After blood sample tubes in a standing position for about 20–30 minutes, for separating serum from the blood cells, we centrifuged at room temperature, 1,500 g for 10 minutes then removed serum very quickly and flash freeze. All serum samples were stored at −80°C before analysis.

### Measurement of Serum β-hCG and Ang-2

Serum Ang-2 levels were determined using commercially available ELISA kits (R&D Systems, USA). Patient serum samples and commercially available trilevel controls (R & D Systems) were analyzed in duplicate. A standard curve was generated for each run, and interassay and intra-assay coefficients were calculated for quality assurance purposes. In the concentration studied, intra and interassay coefficients of variation were 5.7% and 9.4% for Ang-2 detection. The minimum level of detection for Ang-2 was 8.3 ng/L. Serum concentrations of β-hCG were measured by an electrochemiluminiscence immunoassay (ECLIA) intended for use on the automated analyzer Modular Analytics E170 (Roche Diagnostics, Germany). The results were expressed as IU/L and the lower limit of detection was <0.1 IU/L.

### Statistical Analysis

Results are presented as mean±SD or median and interquartile range (IQR), depending on the distribution. Univariate comparisons of continuous variables were performed using unpaired *t*-test for normally distributed data, or nonparametric Mann-Whitney U-test for non-normally distributed variables. For multiple comparisons of several groups, ANOVA or Kruskall-Wallis test were performed. For comparing categorical data, chi-square test was performed. Furthermore, Spearman's rank correlation coefficient was used to explore the relationship between β-hCG and Ang-2 in the PUB and NUB groups. Sensitivity, specificity, positive predictive values (PPV), negative predictive values (NPV)were estimated. ROC analysis was used to determine the optimum cut-off value for the studied diagnostic markers. Furthermore, when the values for β-hCG and Ang-2 exceeded the above mentioned cut off values for both we calculated the sensitivity, specificity, PPV, NPV.Statistical tests were evaluated at the two-sided 0.05 significance level. All statistical calculations were performed using the SPSS for Windows.

## Results

### Baseline Characteristics

**Figure 1:**
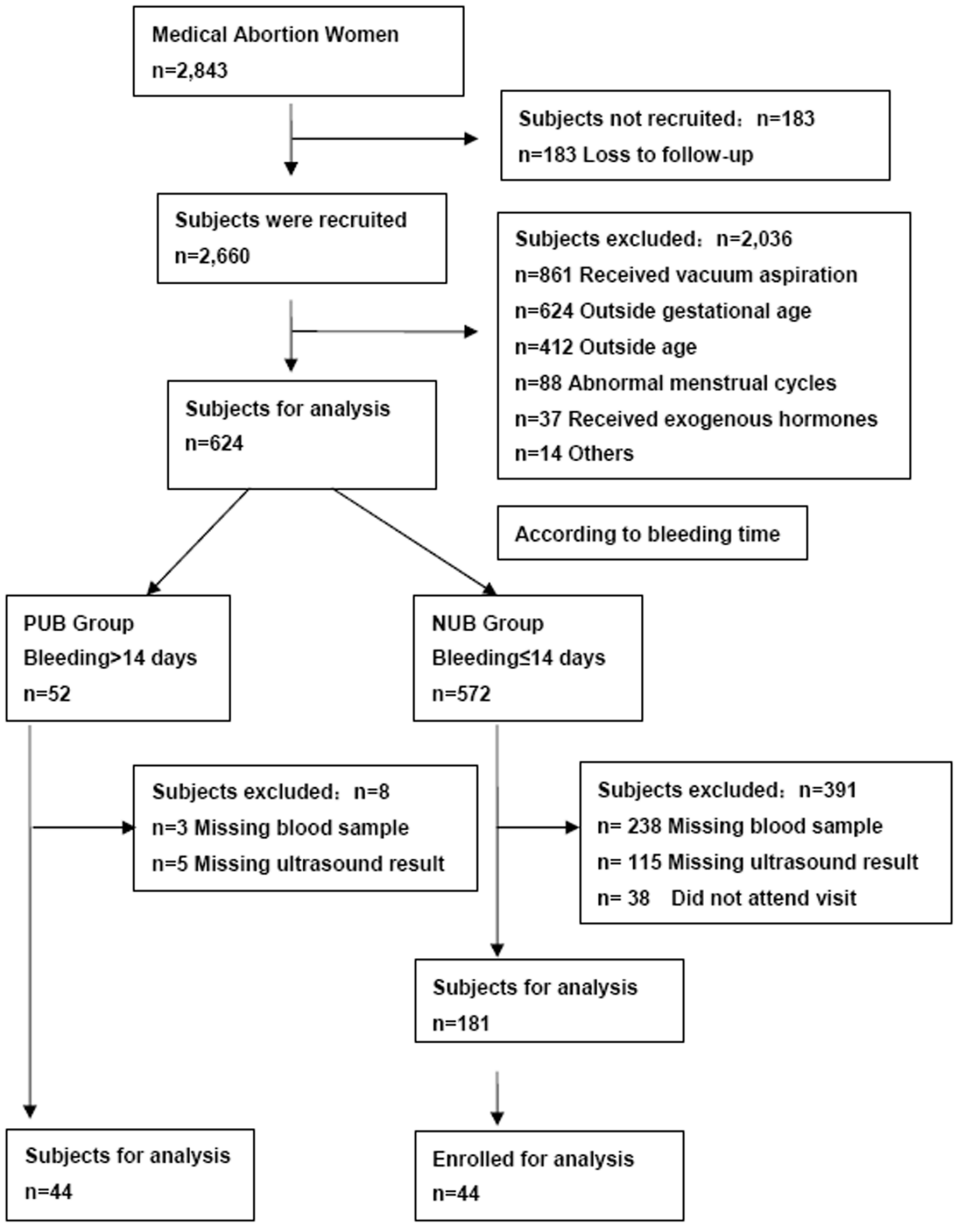
Flow diagram of subjects who underwent medical.

Figure 1 describes the study participants who underwent medical abortion and the reasons why subjects were excluded. In all, women were excluded due to one or more of the following reasons: β-hCG measurements on incorrect days or missing blood sample, performance of ultrasonography on incorrect time or missing ultrasound result, outside gestational age, outside age, abnormal menstrual cycles, received exogenous hormones and not attend visit. The baseline characteristics of women in each group were similar **(** **Table 1** **)**. Overall, no significant difference was found between the two groups with respect to age, gravidity, Maternal BMI, gestational age, menstrual cycle. Only duration of bleeding showed a significant difference between the PUB group and NUB group. All samples were found to contain sufficient material for assay.

**Table 1:** Baseline Characteristics of Women Underwent Medical Abortion.

	PUB Group	NUB Group	p-Value
Maternal age (years ± SD)	27.8±5.3	26.5±4.5	0.15
Gravidity (Primipara) (n [%])	24(54.5%)	22(50.0%)	0.67
Gravidity (Multipara) (n [%])	20(45.5%)	22(50.0%)	
Maternal BMI (Kg/m^2^± SD)	31.4±4.2	30.6±4.6	0.21
Mean gestational age (days)	46.6±4.7	42.3±4.5	0.34
Menstrual cycle (days)	27.1±5.2	28.4±4.1	0.28
Median Serum β-hCG (IU/L) *	53,675(41,131–89,704)	58,396(40,245–94,318)	0.907
Median Serum Ang-2 (ng/L) *	2,574(1,606–3,367)	2,628(1,347–3,590)	0.809
Duration of bleeding (days)	19.6±4.8	9.5±3.6	0.001

BMI: Body mass index;

* Data are given in median and interquartile range (IQR).

### Serum β-hCG and Ang-2 Levels after Medical Abortion

**Figure 2:**
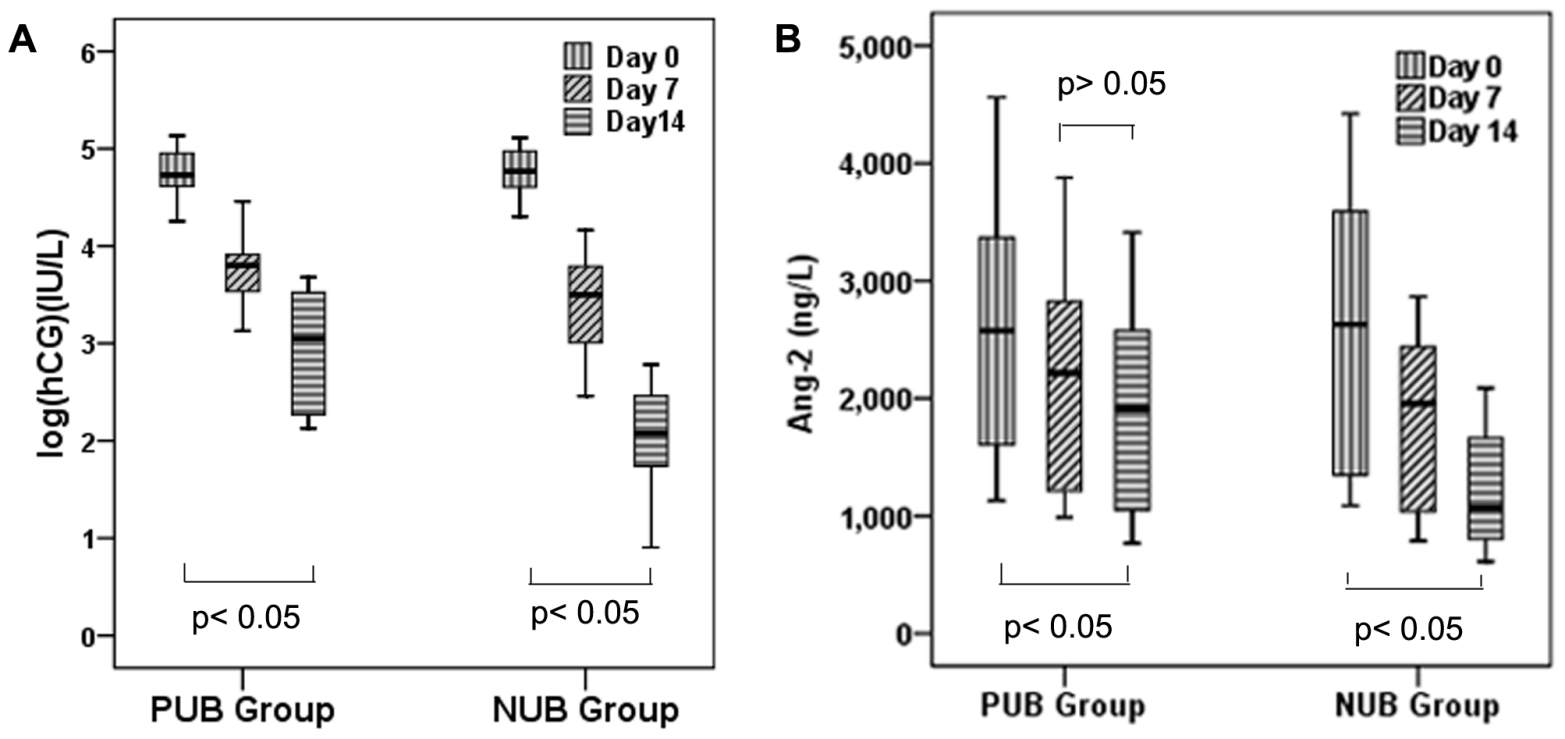
Quantitative Values of Serum β-hCG and Ang-2 After Medical Abortion. The box indicates the lower and upper quartiles and the central line marks the median. The points at the end of the whiskers represent the range of the values.

Serum β-hCG and Ang-2 levels on day 7 and on day 14 after after medical abortion were signifcantly higher in PUB group than in NUB group**(** **Table 2** **)**. Kruskal–Wallis test was employed to compare the serum β-hCG and Ang-2 levels among different days in each group (p<0.05), We proceeded with the Mann–Whitney to see the differences between pairs of days. In this analysis, significant differences of β-hCG levels were found between different days in each group (p<0.05), significant differences of Ang-2 levels were found between different days in NUB group (p<0.05), but there was no difference between day 7 and day 14 after medical abortion in PUB group **(** **Fig. 2** **)**.

**Table 2:** Serum β-hCG and Ang-2 Levels After Medical Abortion.

Day After Medical Abortion	Biomarkers	PUB Group	NUB Group	p-Value
On Day 7	β-hCG(IU/L)	6,314(3,425–8,169)	3,162(1,024–6,158)	0.001
	Ang-2(ng/L)	2,245(1,214–2,826)	1,955(1,036–2,437)	0.035
On Day 14	β-hCG(IU/L)	1,128(187–3,345)	119(55–271)	<0.001
	Ang-2(ng/L)	1,912(1,049–2,543)	1,067(765–1,664)	<0.001

Data are given in median and interquartile range (IQR).

**Figure 3:**
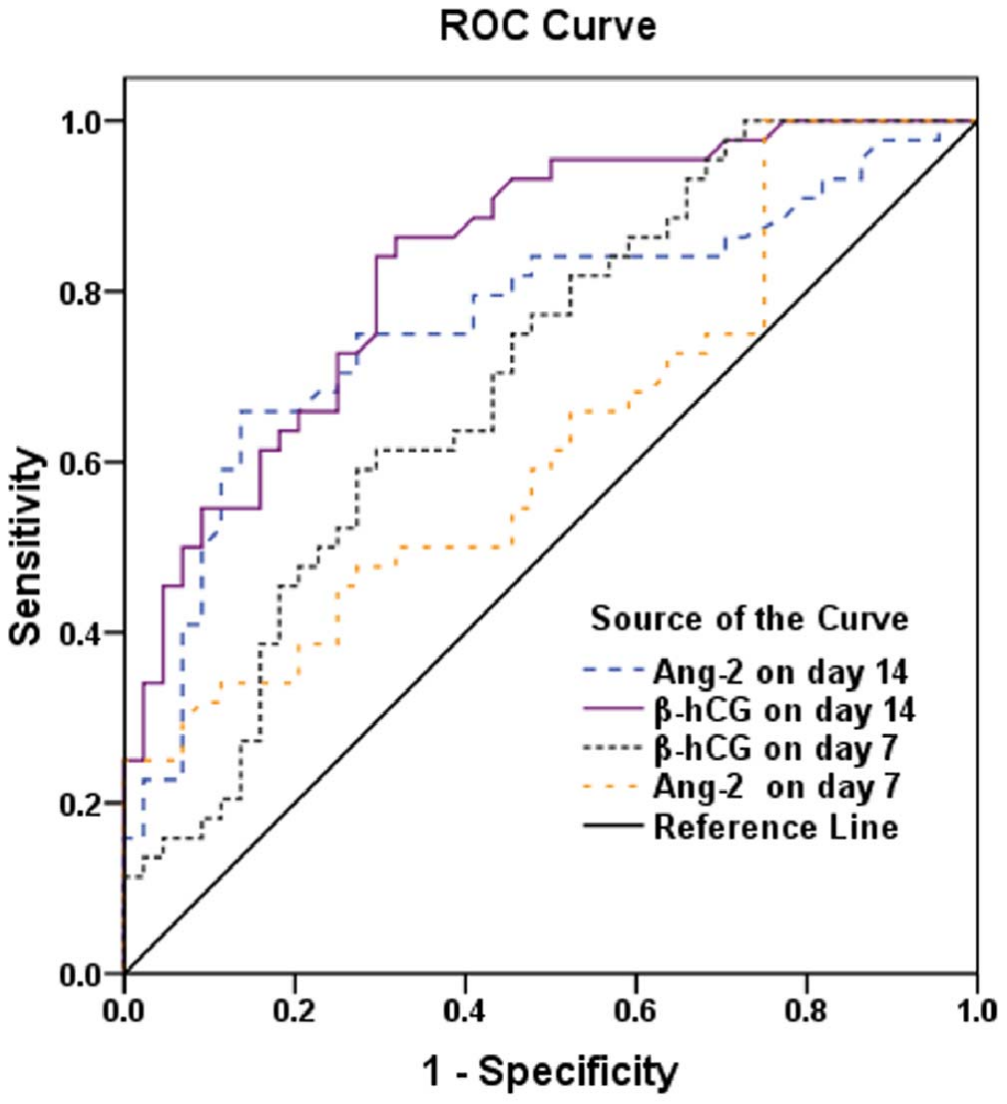
ROC Curve Analyses for the Diagnostic Accuracy of β-hCG and Ang-2 to Discriminate PUB from NUB.

ROC analyses were calculated and plotted for the diagnostic accuracy of serum β-hCG and Ang-2 concentration to discriminate a PUB and a NUB. Plotted as ROC curves **(** **Fig. 3** **)**, β-hCG AUC was 0.65 (95% CI, 0.53–0.76) on day 7, rising to AUC = 0.83 (95% CI, 0.75–0.92) on day 14. Using Ang-2 on day 7 and day 14 as predictive parameter resulted in an analogous AUC (AUC = 0.61 on day 7, AUC = 0.78 on day 14). The sensitivity and specificity for the biomarkers at their respective cut-points are shown in **Table 3**.

**Table 3:** Accuracy of the Biomarkers in Diagnosing PUB on Day 14 After Medical Abortion.

Biomarkers	Sensitivity	Specificity	AUC	P-Value	Cut-Point	PPV	NPV
β-hCG	0.86	0.75	0.83	<0.05	125 IU/L	0.77	0.85
Ang-2	0.75	0.68	0.78	<0.05	1523 ng/L	0.70	0.73
β-hCG+Ang-2	0.64	0.95	–	–	–	0.93	0.72

β-hCG as well as Ang-2 were potential predictors of PUB after medical abortion due to low PPV. We calculated the sensitivity, specificity, PPV, NPV for PUB when the values for β-hCG and Ang-2 exceeded the suggested diagnostic cut off values for both as you can see in row β-hCG+Ang-2 in the Table 3. In comparison PUB and NUB, the combination of β-hCG+Ang-2 has higher PPVs in relation to β-hCG and Ang-2, but it has not the higher NPVs. The corresponding PPV and NPV and AUCs are shown in **Table 3**.

### Correlation between β-hCG and Ang-2

Baseline levels of women underwent a medical abortion, Ang-2 serum levels moderately correlated with serum β-hCG levels with statistically significant correlation coefficients of 0.536. Correlation analysis was also conducted to explore the relationship between β-hCG and Ang-2 in women after medical abortion. On day 7, there is a low positive correlation (ρ = 0.371) without being statistically significant (p-value = 0.165>0.05). On day 14, there is also a low positive correlation (ρ = 0.186) without being statistically significant (p-value = 0.312>0.05).

## Discussion

Medical abortion requires careful monitoring to ensure that the process is complete. Many current medical abortion protocols necessitate extensive involvement of doctors [8]. Although most studies have reported an average duration of bleeding after medical abortion of approximately seven days, bleeding may last for as long as 21 days [5]. In the event of persistent bleeding, surgical intervention should be considered only after careful evaluation of the patient. The suspected late failures in the present study were identified after day 14, reflecting a long and tiring course of bleeding and/or pain. The consequences of overlooking failed uterine evacuation are limited, because the risk of serious morbidity associated with retained tissue is minimal and because failures ultimately will be revealed clinically. angiogenin increased expression in endometrial residue tissue of women after abortion [24], tissue testing is invasive method, can not early effective evaluation of PUB, and need to look for other detection technology to effective evaluation PUB after medical abortion. With this background we conclude that the analyzed variables used as diagnostic tests would lead to a reduced number of unnecessary interventions. Being able to diagnose these PUB earlier would optimize the medical abortion procedure. Followed the patients’ clinical course after a two week follow-up, we were able to analyze the prognostic value of serum β-hCG and Ang-2 levels as predictors of PUB. The overall percentage decline in serum β-hCG is consistent with earlier findings [9], [11]. Both the quantitative values of serum β-hCG and Ang-2 after medical abortion were significantly higher in women who turned out to be PUB than in women whose uterine bleeding were normal.This study provides the data to demonstrate that serum β-hCG and Ang-2 are potential markers of PUB after medical abortion.To our knowledge, this is the first study on serum Ang-2 expression in women with a medical abortion.

The novel data show that serum levels of Ang-2 are increased during the early stage of gestation while serum levels of Ang-1 and sTie2 are relatively stable throughout the gestation [20], [21], [25], [26], [27], [28].the increase in serum level of Ang-2 during first trimester suggests that Ang-2 is required at the early stage of pregnancy. In addition, this increase of Ang-2 in pregnancy serum coincides with the timing of placental vascularisation and development [18]. previous studys reported that maternal serum measurements of angiogenic factors can be used as markers to identify ectopic pregnancies or miscarriage [22].The data presented in this report describing the serum Ang-2 levels may be useful adjuncts for clinical management, especially when vaginal bleeding is prolonged or when Ang-2 levels are much above expected values.

The profile of Ang-2 serum levels during pregnancy exhibits a similar pattern to hCG [29]. Miyabayashi et al [30] showed that Ang-2 mRNA levels in the ovary are increased in response to hCG. Wulff C et al [31] showed that Administration of hCG was associated with an increase in the Ang-2 mRNA area of expression and grain density in individual luteal and endothelial cells. In our study of women with a medical abortion,the Ang-2 values were correlating with β-hCG values raising the expectation that Ang-2 could be β-hCG-independent biomarkers, which consistent with earlier findings could supplement transvaginal ultrasound. But the relationship between β-hCG and Ang-2 in women after medical abortion On day 7 is a low positive correlation (ρ = 0.371) without being statistically significant. On day 14, there is also a low positive correlation (ρ = 0.186) without being statistically significant. Given that there are numerous serum factors, it is difficult for one to identify a specific serum factor that may be involved in the regulation of Ang-2 after medical abortion. Pichiule et al [32]suggests that increases in Ang-2 during hypoxic conditions may be via a COX-2-dependent prostanoids pathway. The aim of the present study was not to explore whether reduction of β-hCG and Ang-2 directly or indirectly relates to the development of PUB. Thus, it is uncertain whether the high levels of β-hCG and Ang-2 in medical abortion women are the consequence of the pathological processes that take place or whether their increase participates in the induction of PUB. The pathophysiological significance of our observations can necessary be studied through serial measurements and further tissue expression assessment of these markers in a larger prospective study.

Both serum β-hCG as well as Ang-2 levels after medical abortion were higher in women who turned out to be PUB. ROC analyses and Plotted as ROC curves, β-hCG AUC was 0.83 (95% CI, 0.75–0.92) on day 14 and Ang-2 AUC was 0.78 (95% CI, 0.68–0.89) on day 14. We found that threshold levels of β-hCG and Ang-2 without high PPV leading not enough to identifcation of PUB patients. As an example: using β-hCG 125 IU/L on day 14 as a threshold, the specifcity was 0.75, the PPV was 0.77, the sensitivity was 0.86. Used in a clinical situation, this means that 23% of performed interventions would be unnecessary, while 14% of the failures would be missed. It should be emphasized that choosing a low β-hCG and Ang-2 cut-off point for clinical intervention would lead to unnecessary surgical treatment for a significant number of patients. Increasing the cut-off would decrease the false positive rate and improve the specificity of the test measurement. we conclude that none of the two variables can be used as perfcet diagnostic tests in predicting PUB after medical abortion. Although our data have been shown to be of discriminatory value, but them were found to be of limited use. The demands for a diagnostic test depend on the clinical situation, the consequences of a positive test result, and the risks connected with an overlooked condition. To avoid unnecessary interventions, the PPV of the applied test must be high. The specifcity should not be <0.95, which is the approximate chance of a successful course using this medical abortion regimen.

It may be that β-hCG and Ang-2 could be included in a combination which may be needed in order create a diagnostic algorithm in order to achieve optimal sensitivity and specificity for the various medical abortion outcomes. Higher PPV were achieved by combining changes in β-hCG and Ang-2, but still at the expense of sensitivity. As an example: a β-hCG value >125 IU/L on day 14 combined with a Ang-2>1523 ng/L on day 14 gave the PPV 0.93, but the corresponding sensitivity was 0.64. Similar values were seen with other combinations of different threshold levels.

## Conclusions

Quantitative values of serum β-hCG and Ang-2 after medical abortion were significantly higher in women who turned out to be PUB than in women whose uterine bleeding were normal. Both parameters are clinically useful as a diagnostic test in predicting PUB after medical abortion, and can be helpful in uncertain clinical situations, but should be considered as supplementary to a general clinical evaluation.
